# Perioperative Outcomes in COVID-19 Obstetric Patients Undergoing Spinal Anesthesia for Cesarean Section: A Prospective Observational Study

**DOI:** 10.3390/healthcare10010023

**Published:** 2021-12-24

**Authors:** Omar Ababneh, Mustafa Alrabayah, Ahmad I. El-Share’, Isam Bsisu, Yara Bahar, Banan Dabousi, Alia Sandoqa, Dania AlWreikat, Ayman Qatawneh

**Affiliations:** 1 Department of Anesthesia and Intensive Care, School of Medicine, The University of Jordan, Amman 11942, Jordan; m.rabayah@ju.edu.jo (M.A.); ahmad92shara@gmail.com (A.I.E.-S.); yarabahar@gmail.com (Y.B.); bdabousi@yahoo.com (B.D.); alia.sandoqa@hotmail.com (A.S.); daniawreikat@hotmail.com (D.A.); 2 Department of Obstetrics & Gynecology, School of Medicine, The University of Jordan, Amman 11942, Jordan; a.qatawneh@ju.edu.jo

**Keywords:** COVID-19, cesarean section, pregnancy, neuraxial anesthesia

## Abstract

Coronavirus disease 2019 (COVID-19) adds more challenges to the perioperative management of parturients. The aim of this study is to examine perioperative adverse events and hemodynamic stability among COVID-19 positive parturients undergoing spinal anesthesia. This prospective observational investigation was conducted at a tertiary teaching hospital in Jordan between January and June 2021, during which 31 COVID-19 positive parturients were identified. Each COVID-19 positive parturient was matched with a COVID-19 negative parturient who received anesthesia under similar operating conditions as a control group. Of the 31 COVID-19 patients, 22 (71%) were otherwise medically free, 8 (25.8%) were emergency cesarean sections. The sensory level of spinal block after 10 min was T8 (T6–T10) among COVID-19 positive group, compared to T4 (T4–T6) among control group (*p* = 0.001). There were no significant differences in heart rate, SBP, DBP, and MAP intraoperatively (*p* > 0.05). Twelve (36.4%) neonates born to COVID-19 positive patients were admitted to NICU, compared to four (11.8%) among control group (*p* = 0.018). There was no statistically significant difference in postoperative complications. In conclusion, spinal anesthesia is considered a safe anesthetic technique in COVID-19 parturients, and therefore it is the anesthetic method of choice for cesarean deliveries among COVID-19 patients.

## 1. Introduction

The whole world has been changing during the last two years after Coronavirus disease 2019 (COVID-19) pandemic emerged and spread all over the world. This global crisis affected all age groups in every single country, causing a huge medical and economic burden. Perioperative management of parturients has always been considered challenging, especially regarding difficult airway and pulmonary complications [1]. COVID-19 infection adds more challenge especially in patients already having pneumonia and acute respiratory distress syndrome (ARDS). Pregnant ladies are more prone to morbidity, intensive care unit (ICU) admission, and mortality due to COVID-19 in comparison with non-pregnant females [2,3], since they have relatively depressed immunity and several cardiopulmonary physiologic changes during pregnancy that can theoretically increase the risk from COVID-19 infection [4].

The main goal of providing anesthesia care to parturients is analgesia, in addition to reducing all potential pulmonary complications. Neuraxial anesthesia remains the best anesthetic method for cesarean section as it spares the airway manipulation risk, and significantly decreases pulmonary complications in comparison with general anesthesia [5]. In COVID-19 patients, it is recommended to perform neuraxial anesthesia for parturients, whether they were delivering normally or via cesarean sections, as it is not expected to worsen the outcomes of COVID-19 patients’ pneumonia [6,7,8]. 

The average daily number of new deliveries in Jordan is 540 deliveries, including normal vaginal deliveries and cesarean sections [9]. Although there is no precise statistics about the rate of COVID-19 infection among this group in Jordan, it is estimated to be high, as the two peaks caused a large outbreak in number of cases [10]. Since most delivery procedures for these patients cannot be postponed until full recovery from COVID-19 and accompanying signs and symptoms, examining these patients for outcomes after COVID-19 infection and exploring safer methods of care during their labor is of great concern.

In this study, we aimed at observing perioperative intraoperative hemodynamic stability and postoperative adverse events amongst COVID-19 positive pregnant ladies undergoing cesarean sections under spinal anesthesia.

## 2. Materials and Methods

### 2.1. Study Design and Ethical Statement

This prospective observational case-control investigation was conducted at Jordan University Hospital (JUH), which is a tertiary teaching hospital located in Amman, the capital of the Hashemite Kingdom of Jordan. Data collection took place between January and June 2021, after obtaining the ethical approval from JUH institutional review board (IRB) committee (number: 442/2021/67). All enrolled patients gave verbal and written informed consent to participate with their data in this investigation, and the data were anonymized, and therefore no patient personal information was or will be disclosed. We adhered to the declaration of Helsinki [11] and Good Clinical Practice (GCP) guidelines [12]. The study was reported in accordance with STROBE statement (https://www.strobe-statement.org/, accessed on 1 July 2021).

### 2.2. Patients’ Recruitment and Data Collection

We enrolled all COVID-19 positive pregnant patients undergoing cesarean section. COVID-19 diagnosis was made via conventional real-time reverse transcription polymerase chain reaction (rRT-PCR) testing. Sampling was based on including all COVID-19 positive cases that meet the inclusion criteria within the predetermined study time-frame, resulting in the inclusion of 31 COVID-19 positive cases. After each COVID-19 positive case, another consenting COVID-19 negative case who received anesthesia on the same day and under similar operating conditions was selected as a control patient. At that time of the pandemic, we had an obstetric operating theater for COVID-19 suspected and confirmed cases, which was prepared to deal with such cases. The other obstetric operating room was prepared to receive COVID-19 negative patients, and we enrolled the first consenting COVID-19 negative case after each positive case to ensure having similar operating conditions. The third operating room was reserved only for life-saving emergencies that were not included in this study.

We designed peri-operative chart to collect patients’ general demographic data and medical history, signs and symptoms, laboratory investigations, vital signs; including systolic blood pressure (SBP), diastolic blood pressure (DBP), mean arterial pressure (MAP), heart rate, electrocardiogram (ECG) findings, respiratory rate, end tidal carbon dioxide (ETCO2), oxygen saturation (O2 saturation), temperature, and O2 requirements. In addition, we documented intraoperative fluids management, dosage of intravenous carbetocin and oxytocin after delivering, duration of procedure, occurrence of perioperative adverse events, duration of block, length on hospital stay, and neonatal outcomes (Apgar score, temperature, and admission to the neonatal intensive care unit (NICU)).

### 2.3. Inclusion and Exclusion Criteria

#### 2.3.1. Inclusion Criteria

For COVID-19 positive cases, we included all consenting laboring patients who tested positive for COVID-19 via conventional rRT-PCR testing were enrolled in this study. Patients aging more than 18 years undergoing cesarean section under neuraxial anesthesia, either electively or emergently, were recruited. For control group, we enrolled the first consenting COVID-19 negative parturient after each COVID-19 positive case. All of these control cases underwent cesarean section under neuraxial anesthesia, either electively or emergently.

#### 2.3.2. Exclusion Criteria

We excluded non-consenting patients, patients aging below 18 years, patients with coagulopathy, patients with neuromuscular and neurologic diseases, patients with peripartum cardiomyopathy, and patients who did not receive neuraxial anesthesia. It is noteworthy to mention that none of the patients refused enrollment or withdrew from the study.

### 2.4. Neuraxial Procedure

Standard American Society of Anesthesiologists (ASA) monitoring was applied, 2 intravenous accesses were secured, and 500 mL of lactated Ringer’s solution was given prior to anesthetic procedure. Standard aseptic technique was applied with patient positioned in sitting position. Using midline approach at lower lumbar level, a senior anesthesia resident physician located the L3–L4 lumbar space and then spinal anesthesia was conducted using a 25-gauge pencil point needle, using 10 mg of hyperbaric bupivacaine 0.5% and 20 µg fentanyl, after which a right lumbar–pelvic wedge was applied and patient was positioned and tested for sensory and motor levels prior to commencing the surgical intervention for cesarean delivery patients. No sedation was given during or after labor.

The patient kept her surgical face mask on, and no oxygen was delivered except for those whose oxygen desaturation was below 94%, according to our institutional COVID-19 guideline [13]. After delivery, standard routine obstetric care was maintained. We then recorded the time needed for full reversal of neuraxial blockade, and the timing of the requirement of further post-operative rescue analgesia. 

All medical staff including anesthesia team wore level 3 personal protective equipment (PPE), including liquid-proof apron, N-95 mask, goggles, visor, and overshoes, according to the ASA and Centers for Disease Control and prevention (CDC) COVID-19 protection guidelines [14,15]. Medical staff were then tested for COVID-19 infection via rRT-PCR 72 h after the procedure. 

### 2.5. Postoperative Follow-Up

On the second day, patients were followed-up regarding vital signs stability, adequacy of pain relief and analgesics need, assessment of lower limbs neurological and motor function, nausea, vomiting and pruritis. We inquired about post dural puncture headache (PDPH), which was then managed using bed rest, maintaining hydration, and paracetamol was given when needed. Any changes in patients’ oxygen requirements were documented throughout their hospital stay. We also documented postoperative complications, including deep venous thrombosis, pulmonary embolism, surgical site infection, and postpartum hemorrhage. We also documented intensive care unit (ICU) admission, the cause of ICU admission, the duration of admission, and the outcome of these cases. 

### 2.6. Study Outcomes

#### 2.6.1. Primary Outcomes

The primary outcome of this study is to examine the incidence of intraoperative and postoperative adverse events and maternal morbidity. This includes the comparison between COVID-19 positive and negative parturients in terms of hemodynamic instability and postoperative adverse events. In addition, we investigated the rate of respiratory adverse events, including hypoxia, need for oxygen therapy, ICU admission, need for endotracheal intubation and mechanical ventilation, as well as perioperative cardiac arrests and mortality rate. 

#### 2.6.2. Secondary Outcomes

We reported the success rate and number of trials for neuraxial block, in addition to the level and duration of sensory block, the need for ephedrine and its dosage, and intraoperative blood loss. We also investigated the need to conversion to general anesthesia during cesarean sections and occurrence of obstetric complications. Neonatal Apgar score, morbidity and mortality were also explored. 

### 2.7. Statistical Analysis

Microsoft Excel 2007 software (Microsoft Corp., Redmond, WA, USA) was used for data entry, and then the data were migrated to SPSS software version 25 (SPSS Inc., Chicago, IL, USA). For data analysis, we began data validation, which was carried out by two investigators independently. The data were tested for normality of distribution using Kolmogorov–Smirnov test. The final sample’s data, features, and characteristics were summarized using mean ± SD for normally distributed continuous variables and comparison was made by Student’s *t*-test, while median and interquartile range (IQR) were used for non-normally distributed continuous variables and comparisons were made by Mann–Whitney U test. We used percentages and numbers for presentation of categorical variables, and comparison was made by chi-Squared test. We applied univariable binomial regression analysis to investigate factors associated with NICU admission, and significant values were included in multiple binomial regression model. The results were presented with adjusted odds ratios (OR) with 95% confidence intervals (95% CI). A *p*-value of less than 0.05 was set for statistical significance for all aforementioned statistical tests.

## 3. Results

Overall, 62 parturients were included, of which 31 patients were COVID-19 positive, while the remaining patients in the control group were COVID-19 negative. The mean age of the studied population was 32.8 ± 4.8 years. 38 (61.3%) of the whole sample did not have chronic medical illnesses, while gestational diabetes, chronic HTN, and thyroid disease were the most common medical illnesses, with no difference among the two groups. Only three patients had chronic respiratory disease (asthma). The demographic factors of the studied patients, along with their medical history and laboratory investigations are represented in Table 1, while presenting signs and symptoms of COVID-19 among the 31 COVID-19 positive parturients are demonstrated in Table 2. 

**Figure 1:**
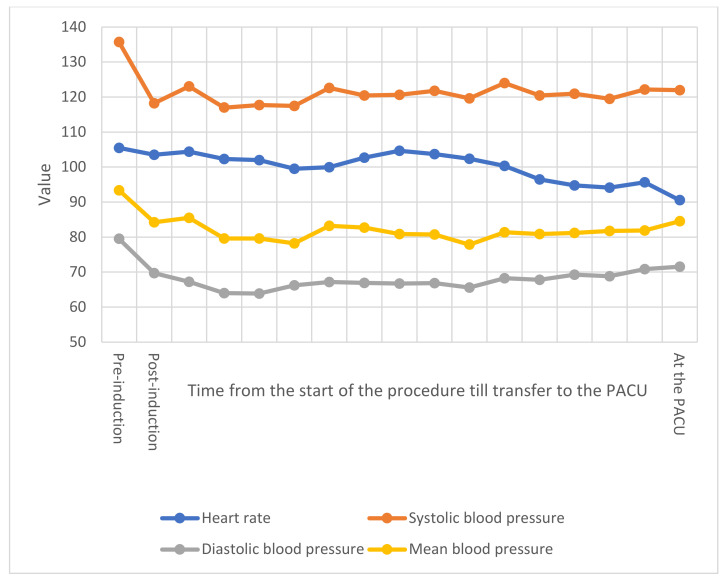
Vital signs in COVID-19 positive patients. The *x*-axis represents the timing of vital signs obtainment. Intraoperatively, vital signs were documented every 5 min until transfer to the PACU. The *y*-axis represents the value of vital signs, measured in beats per minute (BPM) for heart rate, mmHg for systolic, diastolic, and mean blood pressure. PACU: post anesthesia care unit. Vital signs are presented along with comparison with COVID-19 negative group in Supplementary Table S1.

**Figure 2:**
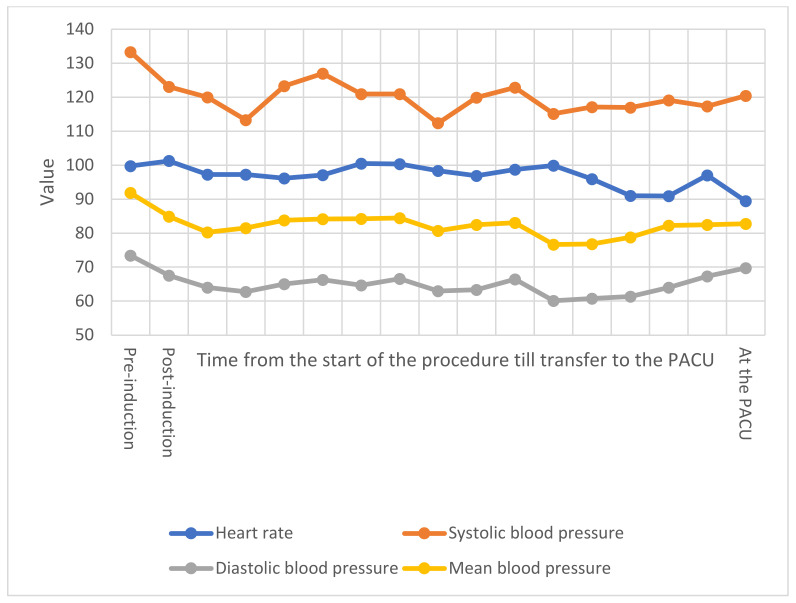
Vital signs in COVID-19 negative patients. The *x*-axis represents the timing of vital signs obtainment. Intraoperatively, vital signs were documented every 5 min until transfer to the PACU. The *y*-axis represents the value of vital signs, measured in beats per minute (BPM) for heart rate, mmHg for systolic, diastolic, and mean blood pressure. PACU: post anesthesia care unit. Vital signs are presented along with comparison with COVID-19 positive group in Supplementary Table S1.

**Figure 3:**
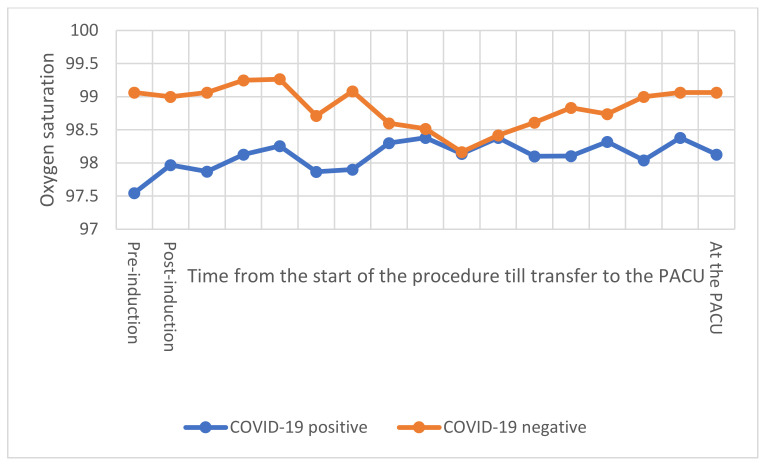
A comparison in oxygen (O_2_) saturation between COVID-19 positive and negative patients. The *x*-axis represents the timing of O_2_ saturation measurement. Intraoperatively, O_2_ saturation was documented every 5 min until transfer to the PACU. The *y*-axis represents the O_2_ saturation in percentage (%). PACU: post anesthesia care unit. Vital signs are presented along with comparison with COVID-19 positive group in Supplementary Table S1.

Cough and myalgia were the commonest presentation (n = 6; 19.4%), followed by sore throat (n = 5; 16.1%), while fever, shortness of breath, tiredness headache, and runny nose were present in 4 (12.9%) of these patients. Of the total 62 cesarean deliveries, 15 were emergency deliveries, with maternal indication being the main indication in 54 (87.1%), while fetal indication was present in only 8 (12.9%) of cases, with no significant difference between the two groups neither in the emergency level, nor in the indication for delivery between the two groups (Table 3). There were no significant differences in the heart rate, SBP, DBP, and MAP intra-operatively (*p* > 0.05) (Figure 1 and Figure 2), while SpO2 was significantly less among COVID-19 positive patients over several intervals (*p* < 0.01) (Figure 3). Ephedrine was administered among 15 (48.4%) in each of the two groups (*p* = 1), with no significant difference in the overall dose of ephedrine per patient, as the average total dose was 6.1 ± 7.9 mg among COVID-19 negative patients and 5.5 ± 7.6 mg among COVID-19 positive patients (*p* = 0.831). There was no significant difference in the duration of cesarean section (*p* = 0.586), nor in blood loss (*p* = 0.256) or amount of crystalloids given intraoperatively (*p* = 0.08). None of the included patients required blood products intraoperatively.

The main neonatal outcomes are represented in Table 4. There were 33 neonates for COVID-19 positive patients (two twins among the 31 cesarean sections), while there were 34 neonates among the control group (one twins and one triplets). We found significant difference between the two groups in NICU admission (*p* = 0.018), with 12 (36.4%) of the neonates born to COVID-19 positive patients being admitted to the NICU, compared to 4 (11.8%) among control group. Multivariable binomial regression analysis for factors associated with NICU admission was performed, the model was overall significant (*p* < 0.001), and it showed that gestational age (OR: 0.39; 95% CI: 0.23–0.66; *p*= 0.001) and COVID-19 positivity (OR: 7.81; 95% CI: 1.11–54.94; *p* = 0.039) were independently associated with NICU admission (Table 5). 

At the post anesthesia care unit (PACU), the level of dermatological block also showed a significant difference between the two groups (*p* < 0.001), but this correlation was contrariwise to the intraoperative levels of sensory block, since the median level of sensory block was T8 (T6–T10) in COVID-19 positive patients, compared to T10 (T10–T10) in control group. There were no statistically significant differences in the time needed for motor block reversal (*p* = 0.26), neither in the time lapsed prior to the need for the first dose of rescue analgesia (*p* = 0.191). There were no significant differences in the heart rate, SBP, DBP, and MAP (*p* > 0.05) (Figure 1 and Figure 2). All patients received 100 mcg Carbetocin and 5 IU Oxytocin IV just after delivering their neonates, and there were no significant differences between the two groups in terms of heart rate (*p* = 0.18), SBP (*p* = 0.242), DBP (*p* = 0.925), and MAP (*p* = 0.278). As presented in Table 6, there were no significant differences between the two groups in any of the postoperative complications (*p* > 0.05). The postoperative follow-up showed that none of the included patients developed deep venous thrombosis or pulmonary embolism. There were two cases of ICU admission (6.5%; *p* = 0.492); one case of postpartum hemorrhage (PPH) in a COVID-19 positive patient, who was later on re-transferred to floor after 2 days. The other case was admitted to the ICU for severe ARDS few days before delivery, she underwent cesarean section under spinal anesthesia on high-flow nasal cannula (HFNC), and couple of days later she was electively intubated after being unstable and due to the failure of other methods to maintain her oxygenation. This patient passed away on the fourth postoperative day, marking a mortality rate of 3.2% among COVID-19 positive parturients (*p* = 1). None of the medical personnel involved in these procedures tested positive for COVID-19 infection via rRT-PCR 72 h postoperatively.

## 4. Discussion

Neuraxial anesthesia is the method of choice for delivering anesthesia and analgesia for laboring women due to the increased risk of maternal morbidity and mortality of general anesthesia [16,17]. Adding to the inherent airway risk of maternal patients, COVID-19 adds special risk for the patient in increasing the risk of developing rapidly deteriorating respiratory complications, especially for symptomatic patients [18], in addition to the risk of transmission of COVID-19 to the anesthesia provider, since airway manipulation is considered a high risk aerosolization procedures that may lead to high viral load transmission [19]. It is recommended to perform neuraxial anesthesia for COVID-19 positive patients when there is no contraindication [6]. Previous reports showed safety of neuraxial anesthesia for patients, and minimal viral transmission to anesthesia personnel who wore PPEs [20,21].

In this prospective observational study, we assessed and evaluated neuraxial anesthesia in 31 COVID-19 positive parturients versus 31 non COVID-19 patients undergoing cesarean sections during the epidemic period in Jordan. Firstly, obstetric outcomes were similar between COVID-19 positive group and control group. It also appeared that COVID-19 infection did not affect hemodynamic parameters of patients undergoing caesarean delivery. Moreover, complication rates were comparable with the control group, with no statistically significant differences.

All patients had adequate sensory block level for the whole cesarean section time; although, remarkably, dermatomal level of neuraxial block at the PACU was significantly higher in COVID-19 positive group in comparison with control group, although no significant difference was found intraoperatively, even though both groups underwent the same neuraxial technique, medication doses, and it was performed by the same anesthesia provider level. The difference in the sensory levels might be caused by delayed onset of neuraxial block in COVID-19 positive patients for an unidentified reason, altered pharmacokinetics, physiological, and inflammatory changes. Therefore, we encourage future studies to document the level of sensory dermatomal block at different time intervals in order to have a better understanding of the change in dermatomal level over time perioperatively.

Both groups of patients showed similar hemodynamic parameters; there was no significant differences among the two groups regarding systolic, diastolic, mean arterial pressure, oxygen saturation, and heart rate. No clinically significant hypotension below 90 mmHg systolic pressure occurred in either group. Although previous literature on non-COVID-19 laboring women indicated that spinal aesthesia is associated with post-spinal hypotension [22], no vigorous comparison was performed to compare between COVID-19 and non-COVID-19 parturients. In their recent study, Zhang et al., concluded that there is an increased risk of neuraxial anesthesia-related hypotension in COVID-19 parturients undergoing cesarean delivery (57.4% in COVID-19 parturients and 41.9% control group) [23]. Although this article provides interesting multicentric data of 101 COVID-19 parturients from three hospitals of Hubei Province, China, the multicentric retrospective design of their study did not permit the inclusion of data regarding pre-operative fluid preloading, nor did the study include information regarding the agents and doses used for spinal anesthesia, the utilization of right lumbar–pelvic wedge upon patients positioning, intraoperative blood loss, and intraoperative dose of oxytocin and carbetocin. In our study, all patients received 100 mcg carbetocin and 5 IU oxytocin IV just after delivering their neonates. Several protective measures help in decreasing the risk for post-spinal hypotension, such as the use of right lumbar-pelvic wedge, decreasing the dose of intrathecal local anesthetic, preoperative fluid preloading, as well as decreasing the dose of intraoperative intravenous oxytocin, which was given with carbetocin after delivering the neonate.

The neonatal APGAR score was similar in both groups after 1, 5, and 10 min of delivery. Despite this, there were more neonatal admissions to NICU in the COVID-19 positive group (12 versus 4 NICU admissions). The main indication for admission was prematurity and for close monitoring. Upon reviewing literature, incidence of neonatal COVID-19 infections seems low, and there are only few rare cases of vertical transmission of COVID-19 [24,25]. As most NICU admissions were related to prematurity, and literature shows no increase in premature delivery rates in COVID-19 positive patients [26,27,28], it remains unclear whether other maternal factors related to their COVID-19 infection have a role in increasing premature deliveries or increasing NICU admissions. Thus, focusing studies and research to explore placental, fetal, and maternal changes in COVID-19 positive parturients are highly suggested.

A systematic review and meta-analysis in 2020 including 24 articles and 1100 pregnancies showed high frequency of preterm births and caesarean deliveries in COVID-19 positive patients [29], despite good clinical condition of the mothers and fetuses, and the cause of that is still not clear. Another systematic review of 161 studies including 3985 pregnancies showed increased rate of preterm deliveries in 23% of COVID-19 pregnancies [30].

Regarding post-operative ICU admission; only one case was admitted due to pure COVID-19 respiratory complications, the patient was in severe ARDS one week before delivery, and she underwent cesarean delivery under spinal anesthesia while on HFNC, and she passed away four days after the surgery. The other case of ICU admission was due to PPH due to obstetric cause, and she was discharged home without complications few days later. In comparison, in a study conducted in India on 115 patients, 5 cases were admitted to the ICU, out of which 1 patient (0.87%) died on the seventh postoperative day [31]. On the other hand, in a study including 61 COVID-19 positive cesarean deliveries in Turkey [32], in which 58 (95.1%) underwent spinal anesthesia, 1 case was admitted to the ICU after delivery under spinal anesthesia, she was intubated later on, and received mechanical ventilation for 68 days, after which she was discharged from the ICU 108 days after her admission. Among the three remaining patients who received general anesthesia, one patient was already intubated at the ICU due to respiratory worsening preoperatively, and she died on the tenth day of ICU follow-up. Based on these findings, Karasu et al., concluded that the mortality rate was 1.6% in all patients, 5.0% in symptomatic patients, and 11.1% in patients with pneumonia [32]. In the current study, the only mortality case was mainly related to the patient’s pre-operative respiratory condition. She had severe ARDS few days before delivery and she underwent cesarean section under spinal anesthesia on HFNC. Couple of days later, she was electively intubated, and she passed away on the fourth postoperative day. Since few records of this adverse outcome are present in each study, we encourage future international registry to include all similar unfavorable outcomes in order to investigate risk factors for perioperative maternal mortality.

Despite the fact that most patients undergoing neuraxial anesthesia for cesarean deliveries are anxious, especially among those having their first delivery, the literature shows that patients who underwent general anesthesia had greater odds for having long term postpartum depression [33]. In this study, we did not examine this aspect due to unethical limitation of providing general anesthesia for COVID-19 patients.

Although one large benefit of awake neuraxial anesthesia for the mother and her newborn child in obstetrics is mothers’ early bonding to their newborns; unfortunately, this could not be achieved in COVID-19 positive mothers, in order to minimize risk of transmission of the virus to their newborns. Fortunately, we observed that COVID-19 positive mothers accepted isolation of their newborns away from them for the sake of their newborns’ safety. 

There were no significant differences in perioperative maternal complications between COVID-19 and non-COVID-19 pregnant women. Upon investigating respiratory adverse events, only one patient developed respiratory function deterioration. She was in ARDS and on non-invasive ventilation prior to her cesarean delivery, and she was managed with spinal anesthesia and remained in her condition intra- and postoperatively, but deteriorated during her ICU stay and died on her fourth postoperative day. In the light of aforementioned information, spinal anesthesia can be considered safe for COVID-19 patients undergoing cesarean deliveries.

This study had several limitations. Firstly, it was a single-centered study including COVID-19 positive cases within a predetermined time-frame. Multicentered national and international investigations on parturients undergoing neuraxial anesthesia are highly recommended, in order to investigate maternal and neonatal outcomes more broadly, especially that the safety of spinal anesthesia in COVID-19 patients is a unique challenge [34]. Secondly, this study did not focus primarily on neonatal outcomes, but rather compared between COVID-19 and non-COVID-19 parturients regarding the safety of spinal anesthesia for cesarean deliveries. Thirdly, the number of attempts of dural puncture must be compared in future studies among anesthesia providers from different training levels to investigate whether the utilization of level 3 PPEs increased the number of unsuccessful attempts for spinal anesthesia. Furthermore, this study was not designed to perform long-term follow-up on patients and their newborns. Lastly, we did not compare the sample to outcomes of general anesthesia, which is justified by the very little number of COVID-19 positive patients undergoing general anesthesia due increased risk of general anesthesia and ethical limitations of performing general anesthesia on parturients for the sake of medical research.

## 5. Conclusions

In conclusion, neuraxial anesthesia remains the best anesthetic method for parturients undergoing cesarean delivery, as it spares the airway manipulation risk, and significantly decreases pulmonary complications among this group of patients. COVID-19 infection does not seem to affect hemodynamic parameters of patients undergoing spinal anesthesia for delivery, nor increases the risk for perioperative complications.

## Figures and Tables

**Table 1:** Demographics and pre-operative data.

Characteristics	COVID-19 Negative (*n* = 31)	COVID-19 Positive (*n* = 31)	Total (*n* = 62)	*p*-Value
Age (years)	33.4 ± 4.6	32.1 ± 5.0	32.8 ± 4.8	0.286
Weight (kg)	81.5 ± 12.4	80.7 ± 13.9	81.1 ± 13.1	0.425
Height (cm)	162.5 ± 5.8	161.3 ± 5.6	161.9 ± 5.7	0.390
Medical history				
Medically free	16 (51.6)	22 (71)	38 (61.3)	0.118
Gestational diabetes	6 (19.4)	4 (12.9)	10 (16.1)	0.490
Chronic hypertension	2 (6.5)	2 (6.5)	4 (6.5)	1.000
Thyroid diseases	5 (16.1)	2 (6.5)	7 (11.3)	0.229
Asthma	2 (6.5)	1 (3.2)	3 (4.8)	0.554
Others	3 (9.7)	1 (3.2)	4 (6.5)	0.301
Drugs allergies	0 (0)	1 (3.2)	1 (1.6)	0.313
Hemoglobin	12.1 ± 1.3	11.9 ± 1.4	12 ± 1.4	0.500
Platelets	202.4 ± 54	218.2 ± 71	210.4 ± 63.1	0.573
Sodium	137.5 ± 2.6	137.1 ± 3	137.3 ± 2.8	0.306
Creatinine	0.51 ± 0.15	0.41 ± 0.14	0.46 ± 0.15	0.035

COVID-19: coronavirus disease 2019; kg: kilograms; cm: centimeters; IQR: interquartile range. Values are represented as number (percent), mean ± standard deviation, and median (IQR).

**Table 2:** Presenting signs and symptoms among the 31 COVID-19 positive patients.

Symptoms	Count (*n*)	Percent (%)
Asymptomatic	20	64.5
Symptomatic	11	35.5
Cough	6	19.4
Myalgia	6	19.4
Sore throat	5	16.1
Fever	4	12.9
Shortness of breath	4	12.9
Tiredness	4	12.9
Runny nose	4	12.9
Headache	4	12.9
GI symptoms	3	9.7
Dizziness	3	9.7
Loss of taste	2	6.5
Oxygen therapy	1	3.2

**Table 3:** Operative data and intraoperative events.

Characteristics	COVID-19 Negative (*n* = 31)	COVID-19 Positive (*n* = 31)	Total (*n* = 62)	*p*-Value
Emergency c-Section	7 (22.6)	8 (25.8)	(24.2)	0.767
Indication of c-Section				
Maternal	27 (87.1)	27 (87.1)	54 (87.1)	1.000
Fetal	4 (12.9)	4 (12.9)	8 (12.9)	1.000
Number of trials	1 (1–1)	1 (1–1)	1 (1–1)	0.545
Level of block after 10 min of spinal anesthesia (median level of thoracic sensory dermatome (IQR))	T4 (T4–T6)	T5 (T4–T6)	T5 (T4–T6)	0.337
Duration c-Section (minutes)	59.8 ± 12.7	63.1 ± 17.4	61.5 ± 15.2	0.586
Blood loss (mL)	662.9 ± 154.9	679.0 ± 199.1	671.0 ± 177.1	0.256
Fluids given (mL)	2274.2 ± 480.3	2371.0 ± 645.1	2322.6 ± 566.1	0.080

COVID-19: coronavirus disease 2019; c-Section: cesarean section; IQR: interquartile range; mL: milliliters. Values are represented as number (percent), mean ± standard deviation, and median (IQR).

**Table 4:** Neonatal characteristics and main outcomes.

Neonatal Characteristic	COVID-19 Negative (*n* = 34)	COVID-19 Positive (*n* = 33)	Total (*n* = 67)	*p*-Value
Gender				
Female	13 (38.2)	18 (54.5)	31 (46.3)	0.181
Male	21 (61.8)	15 (45.5)	36 (53.7)	
Gestational age (weeks)	38.1 ± 2.2	37.5 ± 2.5	37.7 ± 2.4	0.257
Temperature (Celsius)	36.7 ± 0.3	36.7 ± 0.3	36.7 ± 0.3	0.99
Apgar score	8 (8–9)	8 (8–8)	8 (8–8)	0.382
NICU	4 (11.8)	12 (36.4)	16 (23.9)	0.018
Nursery	30 (88.2)	21 (63.6)	51 (76.1)	
Emergency	10 (29.4)	9 (27.3)	19 (28.4)	0.846

COVID-19: coronavirus disease 2019; NICU: neonatal intensive care unit. Values are represented as number (percent), mean ± standard deviation, and median (IQR).

**Table 5:** Univariate and multivariate regression analysis for predictors of NICU admission.

Variables	Univariate				Multivariate			
	OR	95% CI for OR		*p*-Value	OR	95% CI for OR		*p*-Value
		Lower	Upper			Lower	Upper	
Gender (male)	1.14	0.37	3.54	0.817	-	-	-	-
Gestational age (weeks)	0.38	0.24	0.62	<0.001	0.39	0.23	0.66	0.001
Temperature (°C)	0.17	0.02	1.32	0.091	-	-	-	-
Apgar score	0.25	0.07	0.88	0.031	0.36	0.10	1.25	0.107
COVID-19 maternal status (positive)	4.29	1.21	15.13	0.024	7.81	1.11	54.94	0.039
Emergency c-Section	3.64	1.11	11.90	0.033	0.83	0.13	5.12	0.838

COVID-19: coronavirus disease 2019; c-Section: cesarean section; °C: Celsius; OR: odds ratio; 95% CI: 95% confidence interval.

**Table 6:** Postoperative follow-up at the post anesthesia care unit and during the in-hospital stay.

Characteristics	COVID-19 Negative (*n* = 31)	COVID-19 Positive (*n* = 31)	Total (*n* = 62)	*p*-Value
Level of dermatological block at PACU (median level of thoracic sensory dermatome (IQR))	T10 (T10–T10)	T8 (T6–T10)	T10 (T8–T10)	<0.001
Motor Block time (minutes)	118.6 ± 40.9	120.4 ± 83.1	119.5 ± 64.6	0.260
Time for rescue analgesia (minutes)	262.8 ± 185.0	224.7 ± 189.0	240.8 ± 186.5	0.191
Motor function full reversal	31 (100)	31 (100)	62 (100)	N.A.
Nausea	3 (9.7)	1 (3.2)	4 (6.5)	0.612
Pruritus	1 (3.2)	1 (3.2)	2 (3.2)	1
Erythema	3 (9.7)	1 (3.2)	4 (6.5)	0.612
Tenderness	1 (3.2)	4 (12.9)	5 (8.1)	0.354
Swelling	1 (3.2)	0 (0)	1 (1.6)	1
Discharge	0 (0)	0 (0)	0 (0)	N.A.
Temporary peripheral neurological changes	2 (6.5)	0 (0)	2 (3.2)	0.492
Post-dural punctural headache	4 (12.9)	1 (3.2)	5 (8.1)	0.354
Perioperative ICU admission	0 (0)	2 (6.5)	2 (3.2)	0.492
Postoperative mortality	0 (0)	1 (3.2)	1 (1.6)	1

COVID-19: coronavirus disease 2019; PACU: post anesthesia care unit; ICU: intensive care unit. Values are represented as number (percent), mean ± standard deviation, and median (IQR).

## Data Availability

The data presented in this study are available on request from the corresponding author.

## Supplementary Materials

The following are available online at https://www.mdpi.com/article/10.3390/healthcare10010023/s1, Table S1: a comparison between COVID-19 positive and negative patients in perioperative vital signs.
