# Supplementary material for: Perioperative Outcomes in COVID-19 Obstetric Patients Undergoing Spinal Anesthesia for Cesarean Section: A Prospective Observational Study
Source: Healthcare (Basel). 2021 Dec 24;10(1):23. doi: 10.3390/healthcare10010023 (PMC8774980; doi:10.3390/healthcare10010023)
Supplement: Supplementary file 1 [file healthcare-10-00023-s001.zip › healthcare-1461767-supplementary.pdf]

Table S1. A comparison between COVID-19 positive and negative patients in perioperative vital signs.

Characteristics	COVID-19 negative (n=31)	COVID-19 positive (n=31)	p-value
	**Heart rate**		
**Pre-induction**	99.8	105.5	0.310
**Directly post-induction**	101.3	103.5	0.512
**5 minutes**	97.3	104.4	0.300
**10 minutes**	97.2	102.3	0.234
**15 minutes**	96.1	102.0	0.180
**20 minutes**	97.1	99.5	0.837
**25 minutes**	100.5	100.0	0.729
**30 minutes**	100.3	102.7	0.854
**35 minutes**	98.4	104.7	0.348
**40 minutes**	96.9	103.7	0.270
**45 minutes**	98.7	102.3	0.545
**50 minutes**	99.9	100.3	0.877
**55 minutes**	95.9	96.5	0.575
**60 minutes**	91.0	94.7	0.333
**65 minutes**	90.9	94.1	0.556
**70 minutes**	97.0	95.6	0.672
**At the PACU**	89.4	90.5	0.476
	**Syatolic blood pressure**		
**Pre-induction**	133.3	135.7	0.592
**Directly post-induction**	123.0	118.2	0.578
**5 minutes**	120.0	123.1	0.398
**10 minutes**	113.3	117.0	0.885
**15 minutes**	123.3	117.7	0.242
**20 minutes**	126.9	117.4	0.172
**25 minutes**	120.9	122.6	0.350
**30 minutes**	120.9	120.5	0.945
**35 minutes**	112.4	120.6	0.290
**40 minutes**	119.9	121.8	0.514
**45 minutes**	122.8	119.6	0.610
**50 minutes**	115.1	124.0	0.749
**55 minutes**	117.1	120.4	0.507

**60 minutes**	116.9	120.9	0.548
**65 minutes**	119.1	119.5	0.920
**70 minutes**	117.3	122.1	0.352
**At the PACU**	120.4	122.0	0.464
**Diastolic blood pressure**			
**Pre-induction**	73.5	79.5	0.078
**Directly post-induction**	67.5	69.7	0.414
**5 minutes**	64.0	67.2	0.352
**10 minutes**	62.7	64.0	0.544
**15 minutes**	65.0	63.9	0.925
**20 minutes**	66.3	66.2	0.926
**25 minutes**	64.7	67.2	0.541
**30 minutes**	66.6	66.9	0.853
**35 minutes**	63.0	66.7	0.307
**40 minutes**	63.4	66.9	0.380
**45 minutes**	66.4	65.6	0.785
**50 minutes**	60.1	68.2	0.027
**55 minutes**	60.8	67.8	0.045
**60 minutes**	61.4	69.2	0.092
**65 minutes**	64.0	68.8	0.190
**70 minutes**	67.3	70.8	0.310
**At the PACU**	69.8	71.5	0.526
**Mean blood pressure**			
**Pre-induction**	91.9	93.4	0.382
**Directly post-induction**	84.9	84.2	0.877
**5 minutes**	80.2	85.5	0.145
**10 minutes**	81.5	79.6	0.675
**15 minutes**	83.8	79.6	0.278
**20 minutes**	84.2	78.2	0.151
**25 minutes**	84.3	83.2	0.636
**30 minutes**	84.4	82.7	0.620
**35 minutes**	80.7	80.9	0.953
**40 minutes**	82.5	80.8	0.553
**45 minutes**	83.1	77.9	0.090
**50 minutes**	76.7	81.4	0.406
**55 minutes**	76.8	80.9	0.273
**60 minutes**	78.8	81.2	0.682
**65 minutes**	82.3	81.8	0.867
**70 minutes**	82.5	81.9	0.849

**At the PACU**	82.7	84.5	0.637
	**Oxygen saturation**		
**Pre-induction**	98.2	97.5	0.534
**Directly post-induction**	98.4	98.0	0.159
**5 minutes**	98.6	97.9	0.036
**10 minutes**	98.8	98.1	0.048
**15 minutes**	98.7	98.3	0.030
**20 minutes**	99.0	97.9	0.009
**25 minutes**	99.1	97.9	0.005
**30 minutes**	99.1	98.3	0.006
**35 minutes**	99.1	98.4	0.016
**40 minutes**	99.0	98.1	0.013
**45 minutes**	99.1	98.4	0.021
**50 minutes**	99.3	98.1	0.001
**55 minutes**	99.3	98.1	0.001
**60 minutes**	98.7	98.3	0.162
**65 minutes**	99.1	98.0	0.007
**70 minutes**	98.6	98.4	0.287
**At the PACU**	98.5	98.1	0.174
